# Utility of dual-layer spectral-detector CT imaging for predicting pathological tumor stages and histologic grades of colorectal adenocarcinoma

**DOI:** 10.3389/fonc.2022.1002592

**Published:** 2022-10-03

**Authors:** Weicui Chen, Yongsong Ye, Daochun Zhang, Liting Mao, Lei Guo, Hanliang Zhang, Xiaohua Du, Weiwei Deng, Bo Liu, Xian Liu

**Affiliations:** ^1^ Department of Radiology, The Second Affiliated Hospital of Guangzhou University of Chinese Medicine, Guangzhou, China; ^2^ Taizhou Hospital of Zhejiang Province affiliated to Wenzhou Medical University, Taizhou, China; ^3^ Department of Pathology, The Second Affiliated Hospital of Guangzhou University of Chinese Medicine, Guangzhou, China; ^4^ Clinical and Technical Support, Philips Healthcare, Shanghai, China

**Keywords:** colorectal neoplasms, tomography, X-ray computed, pathology, neoplasm staging

## Abstract

**Objectives:**

To assess the utility of Dual-layer spectral-detector CT (DLCT) in predicting the pT stage and histologic grade for colorectal adenocarcinoma (CRAC).

**Methods:**

A total of 131 patients (mean 62.7 ± 12.9 years; 72 female, 59 male) with pathologically confirmed CRAC (35 pT1-2, 61 pT3, and 35 pT4; 32 high grade and 99 low grade), who received dual-phase DLCT were enrolled in this retrospective study. Normalized iodine concentration (NIC), slope of the spectral HU curve (λHU), and effective atomic number (Eff-Z) were measured for each lesion by two radiologists independently. Intraobserver reliability and interobserver agreement were assessed. The above values were compared between three pT-stage and two histologic-grade groups. The correlation between the pT stages and above values were assessed. Receiver operating characteristic (ROC) curves were calculated to evaluate the diagnostic efficacy.

**Results:**

Intra-class correlation coefficients were ranged from 0.856 to 0.983 for all measurements. Eff-Z [7.21(0.09) vs 7.31 (0.10) vs 7.35 (0.19)], NIC_AP_ [0.11 (0.05) vs 0.15 (0.08) vs 0.15 (0.08)], NIC_VP_ [0.27 (0.06) vs 0.34 (0.11) vs 0.35 (0.12)], λHU_AP_ [1.20 (0.45) vs 1.93 (1.18) vs 2.37 (0.91)], and λHU_VP_ [2.07 (0.68) vs 2.35 (0.62) vs 3.09 (1.07)] were significantly different among pT stage groups (all *P*<0.001) and exhibited a positive correlation with pT stages (*r*= 0.503, 0.455, 0.394, 0.512, 0.376, respectively, all *P*<0.001). Eff-Z [7.37 (0.10) vs 7.28 (0.08)], NIC_AP_[0.20 (0.10) vs 0.13 (0.08)], NIC_VP_[0.35 (0.07) vs 0.31 (0.11)], and λHU_AP_ [2.59 (1.11) vs 1.63 (0.75)] in the high-grade group were markedly higher than those in the low-grade group (all *P*<0.05). For discriminating the advanced- from early-stage CARC, the AUCs of Eff-Z, NIC_AP_, NIC_VP_, λHU_AP_, and λHU_VP_ were 0.83, 0.80, 0.79, 0.86, and 0.68, respectively (all *P*<0.001). For discriminating the high- from low-grade CARC, the AUCs of Eff-Z, NIC_AP_, NIC_VP_, and λHU_AP_ were 0.81, 0.81, 0.64, and 0.81, respectively (all *P*<0.05).

**Conclusions:**

The quantitative parameters derived from DLCT may provide new markers for assessing pT stages and histologic differentiation in patients with CRAC.

## Highlights

- CRAC with higher quantitative parameters was associated with more aggressive characteristics.
- Eff-Z, NIC_AP_, and λHU_AP_ demonstrated moderate positive correlations with the pT stages (*r*= 0.503, 0.455, 0.512, respectively).
- Eff-Z, NIC_AP_, and λHU_AP_ exhibited excellent diagnostic capability for predicting advanced-stage or high-grade CRAC (all AUCs≥0.80).

## Introduction

Colorectal cancer (CRC) is the third most common cancer and the second-largest cause of cancer-related death globally. In recent years, the incidence and mortality rates of CRC have shown an increasing trend in people aged under 50 years (1, 2). Tumor-node-metastasis (TNM) stage and histologic grade are significant predictors of survival for patients with CRC (3, 4). Patients with locally advanced colorectal cancer (pathological T3/T4) had a decreased 5-year survival rate compared with those at the early stage (5). In addition, poorly differentiated CRC shows an increased risk of recurrence and a progressively poor prognosis (3, 4).

The choice of therapeutic strategies for CRC patients3is highly dependent on the preoperative stage and tumor ocation. According to the National Comprehensive Cancer Network (NCCN) Clinical Practice Guidelines, neoadjuvant chemoradiotherapy followed by surgery is the preferred modality for locally advanced rectal cancer (below the peritoneal reflection) (5). Although neoadjuvant chemotherapy is not currently a standard treatment for colon cancer, it still has many potential advantages for T3/T4 tumors, including tumor downstaging, reduction in high-risk features of resected tumors, and achieving R0 (margin negative) resection (6–9). Additionally, the Asian Guidelines recommend central lymphadenectomy in selected T2 and all T3/T4 colon cancers (10, 11). Therefore, the correct preoperative identification of advanced-stage CRC would be valuable for determining the most appropriate treatment decision, particularly for high-risk patients, such as those with poor histologic differentiation.

Various imaging modalities have been used for evaluating tumor stage qualitatively, including endoscopic ultrasonography (EUS), multi-detector row computed tomography (MDCT), and magnetic resonance imaging (MRI). The assessment of tumor stage with EUS is well validated, but EUS has two significant limitations: over-staging T2 tumors and inapplicability to stenotic tumors (12). MDCT has been recommended by many current guidelines due to its rapid scanning and thin slices. However, the diagnostic performance is unsatisfactory for tumor staging between radiological stage and pathological results, with an overall consistent rate of 60% ~70% (13–15). Compared to MDCT, MRI has excellent soft-tissue resolution (16). Nevertheless, it is still rather difficult to differentiate fibrosis-induced desmoplastic reaction (pathological T2) from fibrosis-containing tumor cells (pathological T3) (17, 18). Furthermore, the accuracy of analysis with the above imaging modalities depend on the experience of the radiologists, lacking objective and quantitative indicators.

Recently, dual-layer spectral-detector CT (DLCT) has been developed as a novel imaging technology for characterizing different materials by their energy-dependent attenuation properties. In contrast to dual-source or fast kV switching techniques, DLCT employs two layers of detectors to absorb and differentiate high and low energy simultaneously with perfect spatial and temporal alignment. This detector-based spectral separation technique can keep the data intact and improve the accuracy of energy spectrum analysis without altering clinical workflow or increasing radiation exposure (19, 20). Previous studies have suggested that DLCT can enhance the visualization of colorectal lesions, distinguish intra- and extra-luminal iodine or calcium from ingested material, and improve computed tomography (CT) virtual colonography *via* electronic cleansing (21–23). For instance, Obmann MM et al. showed that DLCT could improve polyp conspicuity and reader confidence in a CT colonography phantom, superior to a conventional 120-KVp CT (21). In Wang and colleagues’ research, iodine concentration (IC) and normalized IC (NIC) derived from DLCT were verified to be helpful in assessing local colonic wall thickening caused by colon neoplasia (22). Spectral data appear to be promising for evaluating the pathological prognostic factors of gastrointestinal tumors and providing a differential diagnosis. We hypothesized that the poorly differentiated or advanced stages CRC may present with relatively higher quantitative parameters derived from DLCT due to the numerous tumor angiogenesis or increased tumor heterogeneity. However, evidence of DLCT’s efficacy in differentiating pathological tumor (pT) stage and histologic grade for CRC is still lacking.

Therefore, this study aimed to explore the correlation between the DLCT quantitative parameters and the prognostic factors in colorectal adenocarcinoma (CRAC), further investigating the diagnostic performance of those parameters in the differentiation of advanced-stage from early-stage and high-grade from low-grade CRAC.

## Materials and methods

### Participants

**Figure 1:**
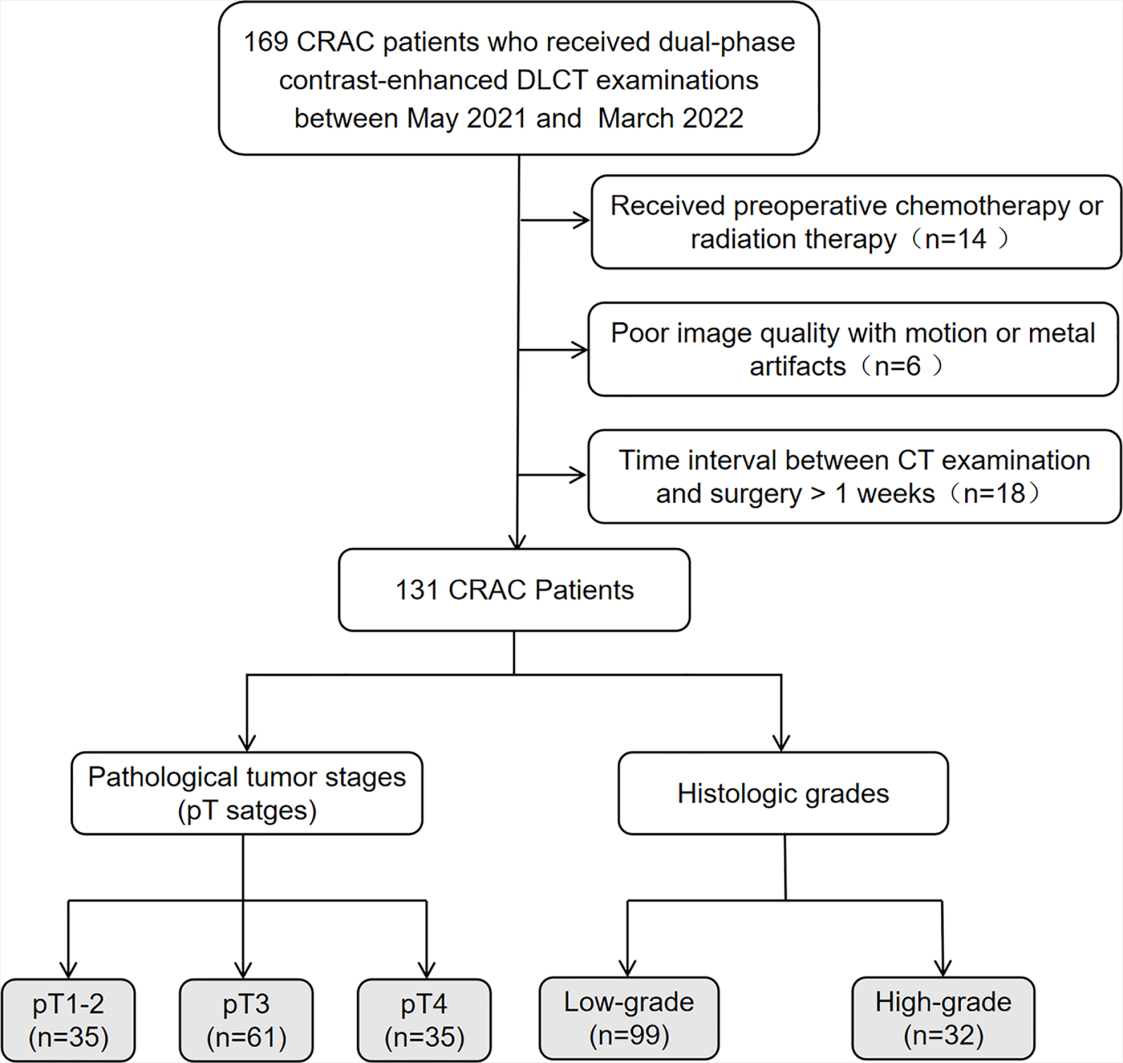
Flowchart of patient selection. CRAC, colorectal adenocarcinoma; DLCT, dual-layer spectral-detector CT.

In this study, 131 CRAC patients demonstrated by pathology were retrospectively enrolled between May 2021 and March 2022. All patients signed informed consent forms according to our institutional guidelines. The inclusion criteria were the following: (a) the presence of tubular adenocarcinoma in the colon or rectum as supported by pathology; (b) complete pathological information, including pT staging and histologic grade; (c) complete clinical information,including carcinoembryonic antigen (CEA) and carbohydrate antigen (CA) 19-9 levels. The exclusion criteria were the following: (a) received preoperative chemotherapy or radiation therapy; (b) poor image quality with motion or metal artifacts; (c) time interval between CT examination and surgery > 1 week. **Figure 1** provides a flowchart showing the patient-selection process.

### Dual-layer spectral-detector CT imaging protocol

CT was performed using a DLCT (IQon spectral CT, Philips Healthcare, Best, the Netherlands) with a nonenhanced and dual-phase contrast-enhanced scan in a craniocaudal direction and the supine position. The patients were injected with a nonionic contrast agent (ultrafast 370, Bayer Healthcare, Guangzhou, China) intravenously by a high-pressure injector at a rate of 2.5 mL/s, with a total dose of 80~120ml (1.5 ml/kg of body weight). Arterial phase (AP) images were triggered by bolus-tracking when the attenuation in the abdominal aorta reached 150 HU. Venous phase (VP) images were acquired 40 seconds after the AP. The scan range comprised the upper edge, including the diaphragm, and the lower, surpassing the symphysis pubis.

The scanning parameters were as follows: tube voltage, 120 kV; tube current, automated modulation with Dose Right Index 22; collimation, 0.6 × 64 mm; rotation time, 0.4 seconds; helical pitch, 1.1.

### Image generation and quantitative analysis

The conventional images and quantitative spectral analysis were performed using IntelliSpace Portal software (Version 10.0; Philips Healthcare). The spectral-based image data were post-processed to generate different image types: (a) iodine density images (iodine map); (b) effective atomic number (Eff-Z) images; (c) virtual mono-energetic images (VMI). All the images were reconstructed with 1mm slice thickness and 1 mm interval.

Two radiologists with more than ten years of experience in gastrointestinal imaging (YY and LM), to whom the clinical and pathological information were not disclosed, evaluated the data independently according to the following steps. First, the 40keV VMI axial images obtained in the arterial and VP were selected to identify tumor margin. Second, a freehand ROI was manually drawn around the edge of the tumor, avoiding fat, necrosis, vessels, and calcification.

The above measurements were performed on each slice of the entire tumor, and the average values of all the ROIs were calculated to minimize the measurement bias. Third, CT values of lesions at 40 and 100keV, and IC of the lesion, abdominal aorta, or external iliac artery were measured on the corresponding spectral images obtained at arterial and VP. The Eff-Z value of tumors was measured on Eff-Z images (pre-contrast phase).

The NIC of tumor and slope of the spectral HU curve (λHU) were calculated at arterial and VP, respectively, according to the following formulas:


$$NIC=\;IC_{tumor}/IC_{artery}$$

λHU= (CT value 40keV—CT value 100keV)/60.

All original measured data were tested for consistency. The final results were expressed as the average values of the obtained data.

### Histopathologic analyses

All patients were operated upon within one week after the DLCT examination. The tumor specimens were further assessed with both HE and immunohistochemical staining by a gastrointestinal pathologist with 12 years of experience in the field (XHD). The evaluation of TNM stages and pathological factors was based on the eighth edition of the American Joint Committee on Cancer Staging system. Tumour grade was classified as grade 1 (well differentiated, greater than 95% gland formation), grade 2 (moderately differentiated, 50%~95% gland formation), or grade 3 (poorly differentiated, less than 50% gland formation). According to two tiered grading system of WHO criteria, the tumors were classified as either low- (G1 and G2) or high-grade CRAC (G3).

### Statistical analysis

Intraobserver reliability and interobserver agreement were determined using the intra-class correlation coefficient (ICC). ICCs are considered to provide adequate reliability if they are higher than 0.75. The normal distribution of quantitative variables was assessed using the Kolmogorov-Smirnov test. Continuous variables are presented as mean ± standard deviation (SD) or median with interquartile range (IQR), as appropriate. The Chi-square test was used to evaluate the enumeration data. The Student’s t-test, one-way analysis of variance (ANOVA), Mann-Whitney U test, or Kruskal-Wallis ANOVA test was used to compare the quantitative parameters between two groups by histologic grades (high and low) and three groups by pT stages (pT1-2, pT3, and pT4). The Bonferroni method was used to correct the *p-*value for multiple comparisons. Spearman correlation analysis was performed to assess the correlation between the pT stages and DLCT parameters quantitatively: weakly correlated: 0~0.40, moderately correlated: 0.41~0.75, strongly correlated: 0.76~1.00. A receiver operating characteristic (ROC) curve was generated to evaluate the diagnostic efficacy of each parameter for differentiating advanced- (pT3/4) from early-stage (pT1/2) and high- from low-grade CRAC. A comparison of ROC curves was applied to test the significance of differences between the area under ROC curves (AUCs).

Statistical analyses were performed using SPSS Statistics 22.0 and MedCalc12.7.2 software. All tests were two sided, and* p*-values lower than 0.05 were considered significant.

## Results

### Comparison of patients’ clinical-pathological characteristics between different pT stages and histologic grades

One hundred and thirty-one patients (male 59, female 72; median age 62.7 ± 12.9 years, range 25~91 years) without distant metastasis were enrolled in the study. According to the postoperative pathological results, the distribution of primary tumor (pT stage) was: pT1-2 (n=35), pT3 (n=61), and pT4 (n=35). Due to inaccurate assessment of preoperative tumor staging, the patients with pT3~4 rectal adenocarcinoma received surgery directly instead of preoperative chemotherapy or radiation therapy. Thirty-two patients had low-grade tumors, and 99 patients had high-grade tumors.

There was a significant difference in the aspect of pN stage among different pT stages and histological grades (all* P*<0.05). CA19-9 and CEA levels markedly varied among different pT stages (all* P*<0.05).

The patients’ clinical-pathological characteristics between different pT stages and histologic grades are shown in **Table 1**.

**Table 1:** Clinical pathological characteristics on 131 CRAC.

Variables	All patients	pT Stages			*P* Value	Histologic Grade		*P* Value
		pT1-2	pT3	pT4		High	Low	
No. of patients	131	35	61	35		32	99	
Age (years), mean ± SD	62.7 ± 12.9	61.7 ± 10.4	61.7 ± 14.0	65.2 ± 130	0.388	60.6 ± 11.7	63.3 ± 13.2	0.306
Gender, No. (%)					0.758			0.061
Female	55.0 (72/131)	51.4 (18/35)	54.1 (33/61)	60.0 (21/35)		40.6 (13/32)	59.6 (59/99)	
Male	45.0 (59/131)	48.6 (17/35)	45.9 (28/61)	40.0 (14/35)		59.4 (19/32)	40.4 (40/99)	
CA19-9 level, No. (%)					<0.001			0.078
Normal	45.0(59/131)	80.0 (28/35)	32.8 (20/61)	31.4 (11/35)		25.0 (8/32)	42.4 (42/99)	
Abnormal	55.0 (72/131)	20.0 (7/35)	67.2 (41/61)	68.6 (24/35)		75.0 (24/32)	57.6 (57/99)	
CEA level, No. (%)					<0.001			0.467
Normal	42.0 (55/131)	88.6 (31/35)	29.51 (18/61)	17.1 (6/35)		31.3 (10/32)	38.4 (38/99)	
Abnormal	58.0 (76/131)	11.4 (4/35)	70.5 (43/61)	82.9 (29/35)		68.8 (22/32)	61.6 (61/99)	
Tumor location, No. (%)					0.037			0.247
Right colon	21.4 (28/131)	14.3 (5/35)	19.7 (12/61)	31.4 (11/35)		25.0 (8/32)	20.2 (20/99)	
Left colon	41.2 (54/131)	28.6 (10/35)	45.9 (28/61)	45.7 (16/35)		50.0 (16/32)	38.4 (38/99)	
Rectum	37.4 (49/131)	57.1 (20/35)	34.4 (21/61)	22.9 (8/35)		25.0 (8/32)	41.4 (41/99)	
Upper Rectum	18.3 (24/131)	34.3 (12/35)	13.1 (8/61)	11.4 (4/35)		9.4 (3/32)	20.2 (20/99)	
Middle Rectum	11.5 (15/131)	20.0 (7/35)	11.5 (7/61)	2.9 (1/35)		6.3 (2/32)	18.2 (18/99)	
Low Rectum	7.6 (10/131)	8.6 (3/35)	9.8 (6/61)	8.6 (3/35)		9.4 (3/32)	3.0 (3/99)	
pN stage, No. (%)					0.001			<0.001
pN0	49.6 (65/131)	65.7 (23/35)	52.5 (32/61)	28.6 (10/35)		12.5 (4/32)	53.5 (53/99)	
pN1	25.2 (33/131)	31.4 (11/35)	21.3 (13/61)	25.7 (9/35)		37.5 (12/32)	19.2 (19/99)	
pN2	25.2 (33/131)	52.9 (1/35)	26.2 (16/61)	45.7 (16/35)		50.0 (16/32)	27.3 (27/99)	
pT stage, No. (%)					/			0.686
pT1-2	26.7 (35/131)	/	/	/		12.5 (4/32)	19.2 (19/99)	
pT3	46.6 (61/31)	/	/	/		56.3 (18/32)	52.5 (52/99)	
pT4	26.7 (35/131)	/	/	/		31.3 (10/32)	28.3 (28/99)	
Histologic grade, No. (%)					0.238			/
High	24.4 (32/131)	17.1 (6/35)	31.2 (19/61)	20.0 (7/35)		/	/	
Low	75.6 (99/131)	82.9 (29/35)	68.9 (42/61)	80.0 (28/35)		/	/	

CRAC, colorectal adenocarcinoma; pT, pathological tumor; CEA, carcino-embryonicantigen; CA19-9, carbohydrate antigen 19-9; pN, pathological lymph node.

Staging of tumors was in accordance with American Joint Committee on Cancer TNM classification;grading of tumors was based on the WHO grading criteria.

Normally distributed data were analyzed by Student’s t test or ANOVA, and were expressed as means ± standard deviations.

### Intraobserver reliability and interobserver agreement

The intraobserver reliability and interobserver agreement of DLCT parameter measurement were excellent. The range of 95% confidence interval (CI) for intraobserver reliability were 0.877 to 0.992. The range of 95% CI for intreobserver agreement were 0.739 to 0.983 (**Table 2**).

**Table 2:** Intraobserver reliabilty and interobserver agreement of DLCT parameter measurement.

Parameter	Intraobserver Reliability (ICC, 95%CI)	Interobserver Agreement (ICC, 95%CI)
Eff-Z	0.921 (0.885~0.952)	0.969 (0.937~0.985)
AP		
CT40keV (HU)	0.962 (0.942~0.976)	0.912 (0.826~0.957)
CT100keV (HU)	0.983 (0.978~0.987)	0.890 (0.771~0.947)
IC_tumor_ (ug/ml)	0.980 (0.952~0.992)	0.856 (0.739~0.923)
IC_artery_ (ug/ml)	0.975 (0.937~0.990)	0.934 (0.876~0.956)
VP		
CT40keV (HU)	0.971 (0.929~0.988)	0.954 (0.905~0.978)
CT100keV (HU)	0.948 (0.877~0.980)	0.900 (0.791~0.952)
IC_tumor_ (ug/ml)	0.959 (0.900~0.984)	0.923 (0.850~0.960)
IC_artery_ (ug/ml)	0.975 (0.947~0.992)	0.966 (0.934~0.983)

DLCT, dual-layer spectral-detector CT; ICC, intra-class correlation coefficients; CI, confidence interval; Eff-Z, effective atomic number; AP, arterial phase; IC, iodine concentration; VP, venous phase.

### Comparison of DLCT parameter values between different pT stages and histologic grades

**Figure 2:**
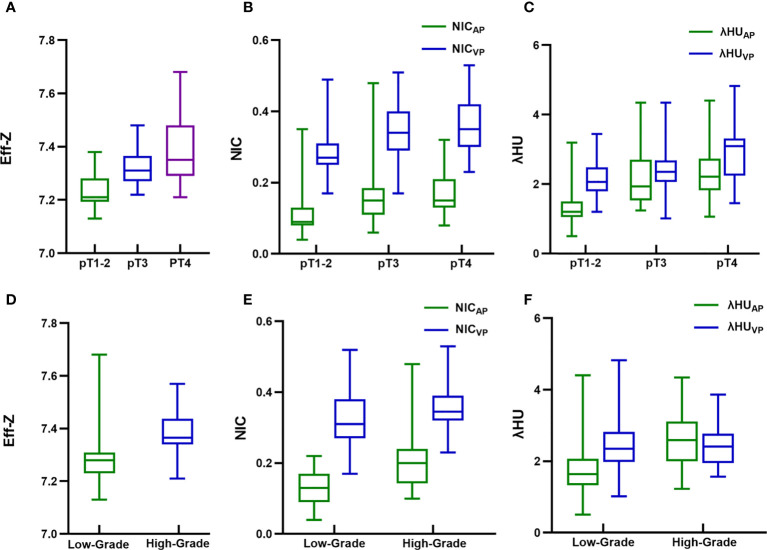
Box and whisker plots of **(A)** effective atomic number (Eff-Z) in pre-contrast phase, **(B)** normalized iodine concentration (NIC) in arterial and venous phase (VP), **(C)** spectral HU curve (λHU) in arterial and VP of different pathological tumor (pT) stage colorectal adenocarcinoma (CRAC); **(D)** Eff-Z in pre-contrast phase, **(E)** NIC in arterial and VP, **(F)** λHU in arterial and VP of high- and low-grade CRAC. Boxes show the upper and lower quartiles, and horizontal lines within boxes indicate median values. Whiskers represent the 95th and fifth percentiles. High- and low-grade CRAC showed no difference in λHU from VP (*P*>0.05), whereas differences in the rest of quantitative parameter values were observed between different pT stages and histologic grades (all *P*<0.05).

**Figure 3:**
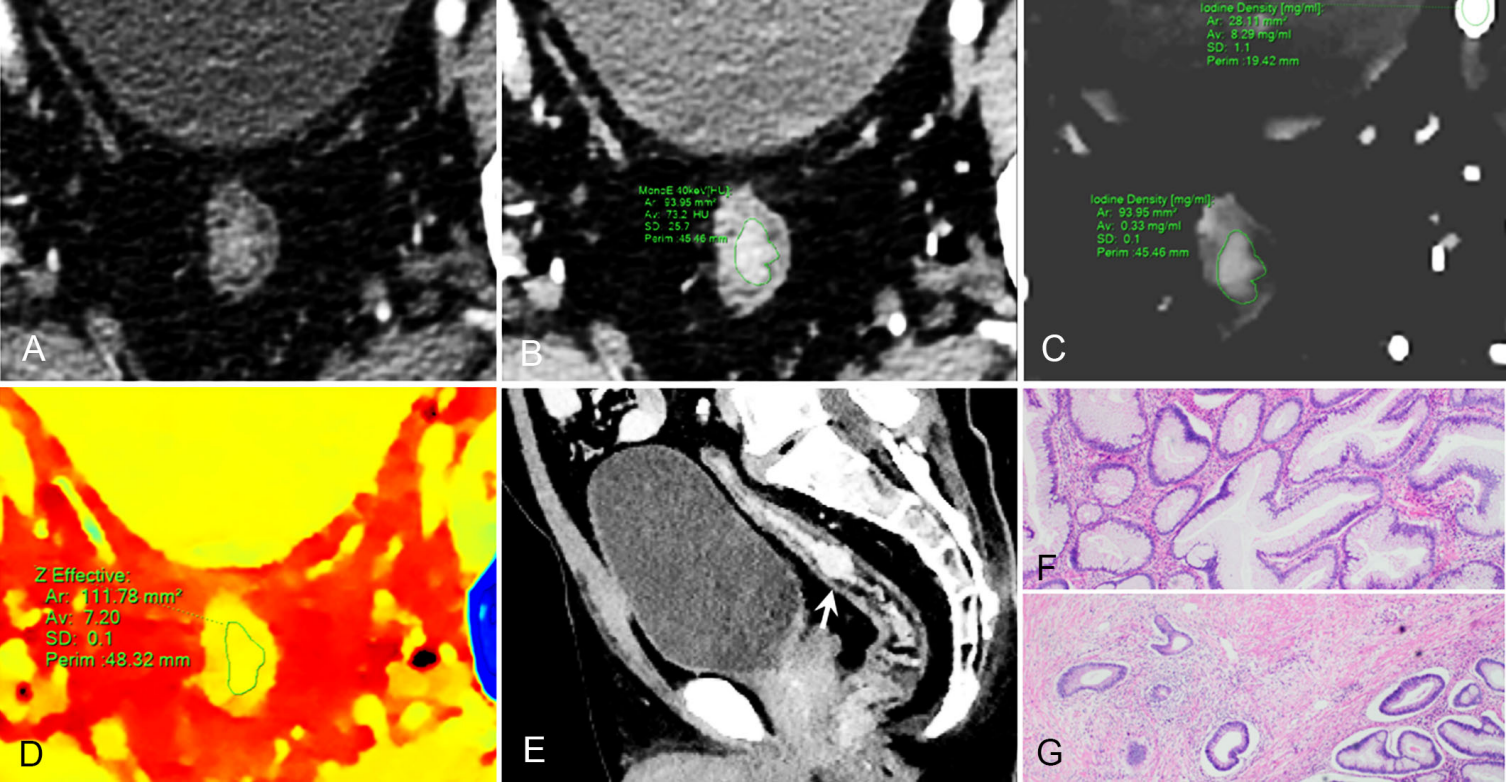
59-year-old man with pathological tumor stage 2 and grade 1 rectal adenocarcinoma, who underwent preoperative dual-layer spectral-detector CT. **(A)** Axial arterial phase (AP) contrast-enhanced image shows irregular wall thickening of the rectum. **(B)** 40 keV virtual mono-energetic image (VMI) in AP shows apparent contrast enhancement between the lesion and surrounding tissue. **(C)** Iodine map in AP shows the lesion with an iodine concentration (IC) value of 0.33 mg/ml; the external iliac artery at the same slice with an IC value of 8.29 mg/ml; a normalized iodine concentration(NIC) value of lesion was 0.04 in AP. **(D)** Effective atomic number (Eff-Z) images in the pre-contrast phase shows the colorful lesion with an Eff-Z value of 7.20. **(E)** Sagittal reconstruction 40 keV VMI shows the tumor located in upper rectum (arrow). **(F)** H E staining demonstrates the tumor is mostly composed of gland-forming elements (×40). **(G)** The tumor cells invade the muscularis propria (×40).

**Figure 4:**
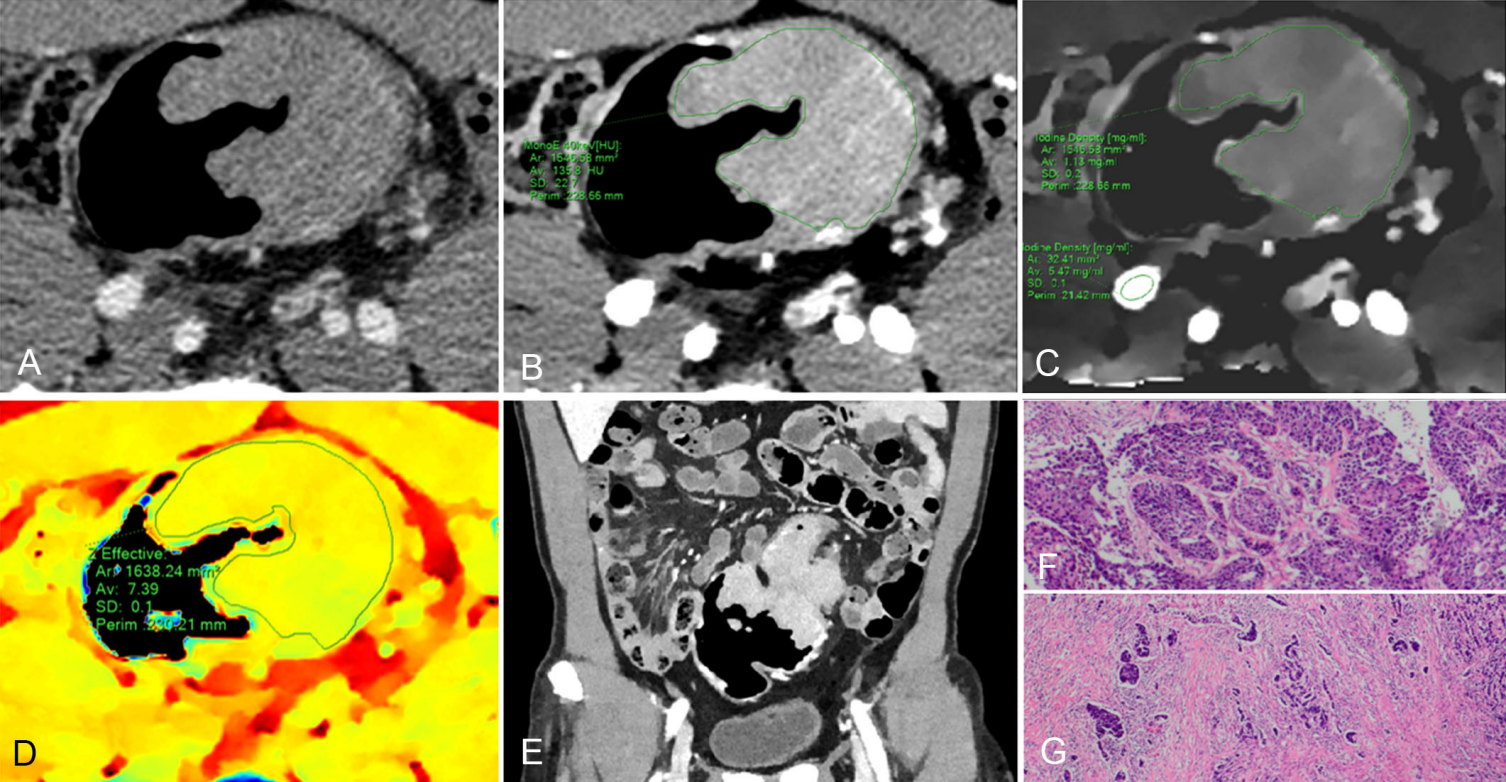
61-year-old man with pathological tumor stage 3 and grade 3 colon adenocarcinoma, who underwent preoperative dual-layer spectral-detector CT. **(A)** Axial arterial phase (AP) contrast-enhanced image shows an irregular mass of the descending colon. **(B)** 40 keV virtual mono-energetic image (VMI) in AP shows apparent contrast enhancement between the lesion and surrounding tissue. **(C)** Iodine map in AP shows the lesion with an iodine concentration (IC) value of 1.13 mg/ml; the external iliac artery at the same slice with an IC value of 5.47 mg/ml; a normalized iodine concentration (NIC) value of lesion was 0.21 in AP. **(D)** Effective atomic number (Eff-Z) images in the pre-contrast phase shows the colorful lesion with an Eff-Z value of 7.39. **(E)** Coronal reconstruction 40 keV VMI shows the tumor located in descending colon. **(F)** H E staining demonstrates the tumor with a minor glandular component (×40). **(G)** The tumor cells invade beyond the muscularis propria (×40).

Based on the Kolmogorov-Smirnov test, all of the quantitative parameters showed non-normal distributions (all *P*<0.05).The Eff-Z values of tumors at the pT1-2, pT3, and pT4 stages were significantly different [7.21(0.09) vs 7.31 (0.10) vs 7.35 (0.19), *P*<0.001, respectively]. The NIC_AP_ and λHU_AP_ values of the tumors were significantly different among pT1-2, pT3, and pT4 stages [0.11 (0.05) vs 0.15 (0.08) vs 0.15 (0.08); 1.20 (0.45) vs 1.93 (1.18) vs 2.37 (0.91), *P*<0.001, respectively]. The NIC_VP_ and λHU_VP_ values of the tumors were significantly different among pT1-2, pT3, and pT4 stages [0.27 (0.06) vs 0.34 (0.11) vs 0.35 (0.12); 2.07 (0.68) vs 2.35 (0.62) vs 3.09 (1.07), *P*<0.001, respectively]. Tumors at the pT4 stage demonstrated higher Eff-Z, NIC_AP_, λHU_AP_, and NIC_VP_ values than pT1-2 tumors, and higher λHU_VP_ values than pT1-2 and pT3 tumors (**Table 3**, **Table S1**, **Figures 2**–**4**).

**Table 3:** Comparison DLCT parameter values between different pT stages and histologic grades, and the correlations with pT stages.

Parameter	pT Stages			*P* Value	*r*	*P* Value	Histologic Grade		*P* Value
	pT1-2 (n = 35)	pT3 (n = 61)	pT4 (n = 35)				High (n = 32)	Low (n = 99)	
Eff-Z	7.21 (0.09)	7.31 (0.10)	7.35 (0.19)	<0.001	0.503	<0.001	7.37 (0.10)	7.28 (0.08)	<0.001
NIC_AP_	0.11 (0.05)	0.15 (0.08)	0.15 (0.08)	<0.001	0.455	<0.001	0.20 (0.10)	0.13 (0.08)	<0.001
λHU_AP_	1.20 (0.45)	1.93 (1.18)	2.37 (0.91)	<0.001	0.512	<0.001	2.59 (1.11)	1.63 (0.75)	<0.001
NIC_VP_	0.27 (0.06)	0.34 (0.11)	0.35 (0.12)	<0.001	0.394	<0.001	0.35 (0.07)	0.31 (0.11)	0.015
λHU_VP_	2.07 (0.68)	2.35 (0.62)	3.09 (1.07)	<0.001	0.376	<0.001	2.40 (0.82)	2.35 (0.84)	0.902

DLCT, dual-layer spectral-detector CT; pT, pathological stage; Eff-Z, effective atomic number; NIC, normalized iodine concentration; AP, arterial phase; VP, venous phase; λHU, slope of the spectral HU curve.

Non-normally distributed data were analyzed by Mann-Whitney U test or Kruskal-Wallis H test, and were expressed as medians (interquartile ranges).

The Eff-Z, NIC_AP_, λHU_AP_ and NIC_VP_ values were significantly different between high- and low-grade CRAC [7.37 (0.10) vs 7.28 (0.08), *P*<0.001; 0.20 (0.10) vs 0.13 (0.08), *P*<0.001; 2.59 (1.11) vs 1.63 (0.75), *P*<0.001; 0.35 (0.07) vs 0.31 (0.11), *P=*0.015]. High- and low-grade tumors showed no difference in λHU_VP_ values [2.40 (0.82) vs 2.35 (0.84), *P*=0.902] (**Table 3**, **Figures 2**–**4**).

### Correlation between DLCT parameters and pT stages

The Eff-Z, NIC_AP_, and λHU_AP_ values demonstrated a moderate positive correlation with the pT stages (*r*=0.503, *P*<0.001; *r*=0.455, *P*<0.001; *r*=0.512, *P*<0.001, respectively). The NIC_VP_ and λHU_VP_ values showed a weak correlation with the pT stages (*r*=0.394, *P*<0.001; *r*=0.376, *P*<0.001, respectively) (**Table 3**).

### Diagnostic performance of Eff-Z, NIC_AP_, λHU_AP_, NIC_VP_, and λHU_VP_ values for discriminating advanced- from early-stage CRAC

**Figure 5:**
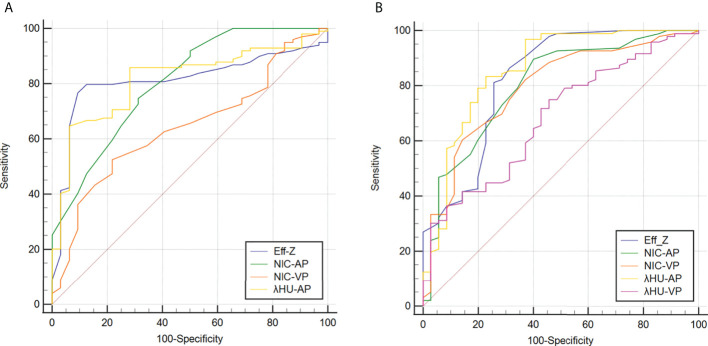
Receiver operating characteristic curves for predicting high-grade **(A)** and advanced-stage and **(B)** CRAC. Details of the area under the curves of each metric are shown in the results section. CRAC, colorectal adenocarcinoma.

For discriminating the advanced- from early-stage CRAC, the AUCs of the Eff-Z, NIC_AP_, and λHU_AP_ values were 0.826 [(95% CI: 0.750~0.887), *P*<0.001], 0.803 [(95% CI: 0.724~0.867), *P*<0.001], and 0.859 [(95% CI: 0.787~0.913), *P*<0.001], respectively. The AUCs of NIC_VP_, and λHU_VP_ were 0.793 [(95% CI: 0.713~0.859), *P*<0.001] and 0.682 [(95% CI: 0.595~0.760), *P*<0.001], respectively. Further pairwise comparisons showed that the AUCs of the Eff-Z, λHU_AP_, and NIC_VP_ values were significantly higher than that of λHU_VP_ (all *P*<0.05) (**Table 4**, **Table S2**, **Figure 5A**).

**Table 4:** Performance of DLCT parameters in differentiating advanced- from early-stage CRAC.

Parameter	AUC	Cutoff	Sensitivity (%)	Specificity (%)	Youden index J	95% CI	*P* Value
Eff-Z	0.826	7.26	81.25	74.29	0.555	0.750~0.887	<0.001
NIC_AP_	0.803	0.10	89.58	60.00	0.496	0.724~0.867	<0.001
λHU_AP_	0.859	1.50	83.33	77.14	0.605	0.787~0.913	<0.001
NIC_VP_	0.793	0.32	60.42	85.71	0.461	0.713~0.859	<0.001
λHU_VP_	0.682	2.10	75.00	54.29	0.293	0.595~0.760	<0.001

CRAC, colorectal adenocarcinoma; AUC, area under curve; CI, confidence interval; Eff-Z, effective atomic number; NIC, normalized iodine concentration; AP, arterial phase; VP, venous phase; λHU, slope of the spectral HU curve.

According to the AUC, the cut-off values of the mean Eff-Z, NIC_AP_, and λHU_AP_ were 7.26 (with 81.25% sensitivity, 74.29% specificity), 0.10 (with 89.58% sensitivity, 60.00% specificity), and 1.50 (83.33% sensitivity, 77.14% specificity), respectively. The cut-off value for the NIC_VP_ and λHU_VP_ were 0.32 (with 60.42% sensitivity, 85.71% specificity) and 2.10 (with 75.00% sensitivity, 54.29% specificity) (**Table 4**, **Figure 5A**).

### Diagnostic performance of Eff-Z, NIC_AP_, λHU_AP_, and NIC_VP_ values for discriminating high- from low-grade CRAC

For discriminating high- from low-grade CRAC, the AUCs of the Eff-Z, NIC_AP_, λHU_AP_ and NIC_VP_ were 0.812 [(95% CI: 0.735~0.875), *P*<0.001], 0.805 [(95% CI: 0.726~0.869), *P*<0.001], 0.815 [(95% CI: 0.738~0.877), *P*<0.001], and 0.643 [(95% CI: 0.555~0.725), *P*<0.001], respectively. Further pairwise comparisons showed that the AUCs of the Eff-Z, λHU_AP_, and NIC_AP_ values were significantly higher than that of NIC_VP_ (all *P*<0.01) (**Table 5**, **Table S3**, **Figure 5B**).

**Table 5:** Performance of DLCT parameters in differentiating high- from low-grade CRAC.

Parameter	AUC	Cutoff	Sensitivity (%)	Specificity (%)	Youden index J	95% CI	*P* Value
Eff-Z	0.812	7.31	90.62	76.77	0.674	0.735~0.875	<0.001
NIC_AP_	0.805	0.16	68.75	74.75	0.435	0.726~0.869	<0.001
λHU_AP_	0.815	1.86	93.75	64.65	0.584	0.738~0.877	<0.001
NIC_VP_	0.643	0.31	78.12	52.53	0.307	0.555~0.725	0.0067

CRAC, colorectal adenocarcinoma; AUC, area under curve; CI, confidence interval; Eff-Z, effective atomic number; NIC, normalized iodine concentration; AP, arterial phase; VP, venous phase; λHU, slope of the spectral HU curve.

The cut-off values of the mean Eff-Z, NIC_AP_, λHU_AP_ and NIC_VP_ were 7.31 (with 90.62% sensitivity, 76.77% specificity), 0.16 (with 68.75% sensitivity, 74.75% specificity), 1.86 (93.75% sensitivity, 64.65% specificity), and 0.31 (with 78.12% sensitivity, 52.53% specificity), respectively (**Table 5**, **Figure 5B**).

## Discussion

Pathological tumor stage and histologic differentiation are the most critical factors for CRAC prognosis (3, 24) and may wanrrant different management approaches (6–11). In this study, we evaluated and compared the relationship between quantitative parameters derived from DLCT and pathological tumor stages and histologic differentiation in patients with CRAC. We found that CRAC with higher quantitative parameters (Eff-Z, NIC_AP_, λHU_AP_, NIC_VP_) was associated with more aggressive characteristics (advanced pT stage, poor histologic differentiation), and exhibited a positive correlation with pT stage. Eff-Z, NIC_AP_, and λHU_AP_ values could successfully distinguish advanced- from early-stage tumors and high- from low-grade CRAC.

To derive the imaging measures, the ROIs were created on each CT slice of the entire tumor. This approach may well reflect the characteristic and heterogeneity of the CRAC. Besides, considering that the physiological distributions of IC in individuals would affect the iodine perfusion (25, 26), we compared the gender and age in the two groups (pT-stage and histologic-grade subgroups) to reduce the bias caused by the two factors. We found no significant differences regarding the gender and age in these two groups.

We observed that the quantitative parameters (Eff-Z in pre-contrast phase, λHU_AP_) increased significantly in the advanced pT stage or high-grade CRAC. Eff-Z, which describes the nature of material or compound interactions with radiation (27, 28), can discriminate and reflect the characteristics of material more accurately than attenuation in HU (29). The Eff-Z of cancerous tissue differs from healthy tissue because of the different trace element concentrations in tumor tissues (30). In most tumors, Eff-Z is consistently higher than healthy tissue, as verified by many studies (31, 32). Additionally, Eff-Z can reflect material heterogeneity. λHU describes the attenuation changes of different materials as a function of the energy spectrum of x-ray photons, which is associated with material composition, density, and interactions between photon energies and material (33, 34). We hypothesized the higher Eff-Z in the plain phase and λHU_AP_ values in aggressive CRAC (e.g., advanced T stage, high grade) may be attributed to increased tumor heterogeneity, where tumor tissue exhibited nuclear pleomorphism and abnormal elemental composition. The spectral curve of the lesion was also affected by the iodinated contrast agent. Higher iodine intake within the tumor leads to a steeper spectrum HU curve slope due to more tissue attenuation of X-rays (35). Besides, it is worth noting that Eff-Z value in the tumor tissue is changed by contrast material after enhancement. The addition to a tumor with a high atomic number element, such as iodine, shifts the linear attenuation coefficients and effective atomic number of that tumor significantly.

Many studies have suggested that IC can reflect blood volume, microvascular permeability, and tissue neovascularization (36–39). In this study, to minimize technical or physiological variabilities, such as injection rate and dose of contrast agent, and individual cardiac output differences, we used NIC values to assess the lesion iodine content. We found that both NIC in AP and VP are significantly higher in the advanced pT stage or high-grade CRAC. According to the acquisition time of our abdomen CT, the AP was triggered by bolus-tracking when the attenuation in the abdominal aorta reached 150 HU, and the VP followed at intervals of 40 seconds. Hence, the AP NIC mainly reflects the degree of neovascularization in CRAC, while the VP NIC represent the microcirculation of the tumor and distribution of contrast media within interstitial spaces (40). Neovascularization is of utmost importance for tumor growth and invasion. Studies have demonstrated an increased micro-vessel density (MVD) in tumors with poor histologic differentiation and poor prognoses (41, 42). MVD is a surrogate marker that reflects tumor angiogenesis and has a significant positive correlation with IC (43). Advanced-stage or high-grade CRAC presents increased angiogenesis and abnormal microvascular permeability, leading to higher NIC values. Our results agreed with those of Cao et al. and Gong et al., who used rapid kV switching dual-energy CT (Discovery 750 HD, GE) to evaluate the colon cancer differentiation degree (44, 45). They showed that the IC and NIC of high-grade colon cancer were significantly higher than that of low-grade colon cancer; Therefore, quantitative parameters of iodine concentration can provide helpful information in distinguishing low- from high-grade colorectal cancer.

We found that the quantitative parameters (Eff-Z in pre-contrast phase, both NIC and λHU in AP) manifested good diagnostic performances in diagnosing pathological tumor stage and histologic grades in CRAC (all the AUCs≥0.80). Notably, we found that the most valuable quantitative parameters were obtained in the AP rather than the VP, in accordance with Li R and colleagues’ results, who found that the NIC value in the AP gave a relatively high diagnostic performance in discriminating poorly differentiated from well-differentiated gastric cancers (46). Previous studies have shown that the AP IC has the strongest correlations with quantitative volume perfusion CT parameters (47), potentially explaining why the AP parameters had the highest discriminating power for differentiating the above pathological factors in CRAC. Conversely, the ability of some metrics to discriminate tumor stages or histologic grades of CRAC was unsatisfactory, such as λHU_VP_ and NIC_VP_, with an AUC of 0.68 and 0.64, respectively. The following two reasons might interpret the results. First, these quantitative parameters obtained in the VP can’t reflect the tumour’s blood supply or heterogeneity accurately. Second, the sample size was relatively small. Expanding the sample size may improve the diagnostic performance of the two parameters.

The present study had some limitations. First, ROI included the entire tumour, but it may not completely match the microscopic histological sample. Second, other rare histologic types were not included, such as mucinous adenocarcinoma, signet ring cell and undifferentiated carcinomas. Future research with more cases of different types is essential to draw broader conclusion. Third, this study was a retrospective and single-center design; hence, we need to perform further prospective clinical trials to validate our results. Finally, because the sample size was imbalanced, the research will be expanded in the future to confirm the accuracy of the results.

In conclusion, DLCT is a potential modality for performing non-invasive evaluations of pT stages and histologic grades in CRAC preoperatively. CRAC with higher values of Eff-Z, NIC_AP_, λHU_AP_, and NIC_VP,_ are associated with more aggressive characteristics and worse prognosis. Eff-Z, NIC_AP_, and λHU_AP_ exhibited excellent diagnostic capability for predicting advanced-stage or high-grade CRAC (all the AUCs≥0.80). The findings indicate that DLCT quantitative parameters may lead to better guidance of surgical and oncological treatment planning for patients with CRAC.

## Data availability statement

The original contributions presented in the study are included in the article/**Supplementary Material**. Further inquiries can be directed to the corresponding author.

## Ethics statement

The studies involving human participants were reviewed and approved by the institutional review board of the second affiliated hospital of guangzhou university of chinese medicine. The patients/participants provided their written informed consent to participate in this study. Written informed consent was obtained from the individual(s) for the publication of any potentially identifiable images or data included in this article.

## Author contributions

WC: Data curation, Writing- Original draft preparation. YY: Writing - Review & Editing. DZ: Data curation, Writing-Original draft preparation. LM: Data curation, Writing-Original draft preparation. LG: Writing- Original draft preparation, Formal analysis. HZ: Data curation, Funding acquisition. XD: Investigation, Resources. WD: Data Curation. BL: Supervision. XL: Conceptualization, Methodology, Writing -Review & Editing. All authors contributed to the article and approved the submitted version.

## Funding

This work was supported by the National Nature Science of Foundation of China[Grant No.82202259] and Youth Talent Project of The Second Affiliated Hospital of Guangzhou University of Chinese Medicine [Grant No. ZY2022YL05].

## Conflict of interest

The authors declare that the research was conducted in the absence of any commercial or financial relationships that could be construed as a potential conflict of interest.

## Publisher’s note

All claims expressed in this article are solely those of the authors and do not necessarily represent those of their affiliated organizations, or those of the publisher, the editors and the reviewers. Any product that may be evaluated in this article, or claim that may be made by its manufacturer, is not guaranteed or endorsed by the publisher.

## Abbreviations

AP, Arterial phase; CRC, Colorectal cancer; CRAC, Colorectal adenocarcinoma; CEA, Carcinoembryonic antigen; CA, Carbohydrate antigen; DLCT, Dual-layer spectral-detector computed tomography; EUS, Endoscopic ultrasonography; Eff-Z, Effective atomic number; IC, Iodine concentration; ICC, Intraclass correlation coefficient; IQR, Interquartile range; MDCT, Multi detector row computed tomography; MVD, Micro vessel density; NCCN, National comprehensive cancer network; NIC, Normalized iodine concentration; ROC, Receiver operating characteristic; SD, Standard deviation; TNM, Tumor node metastasis; VP, Venous phase; VMI, Virtual mono-energetic images; λHU, HU curve.
