# Supplementary material for: Utility of dual-layer spectral-detector CT imaging for predicting pathological tumor stages and histologic grades of colorectal adenocarcinoma
Source: Front Oncol. 2022 Oct 3;12:1002592. doi: 10.3389/fonc.2022.1002592 (PMC9564703; doi:10.3389/fonc.2022.1002592)
Supplement: Supplementary file 1 [file DataSheet_1.docx]

**Table 1** Comparison DSLCT parameter values between different T stages

| **Parameter** | **pT Stages** | | |
| --- | --- | --- | --- |
|  | **pT1-2**  **(n=35)** | **pT3**  **(n=61)** | **pT4**  **(n=35)** |
| Eff-Z | 7.21 (0.09) | 7.31 (0.10) | 7.35 (0.19) |
| T1-2 vs T3 | ＜0.001 | | |
| T1-2 vs T4 | ＜0.001 | | |
| T3 vs T4 | 0.214 | | |
| *P* Value | ＜0.001 | | |
| NIC_AP_ | 0.11 (0.05) | 0.15 (0.08) | 0.15 (0.08) |
| T1-2 vs T3 | ＜0.001 | | |
| T1-2 vs T4 | ＜0.001 | | |
| T3 vs T4 | 0.507 | | |
| *P* Value | ＜0.001 | | |
| λHU_AP_ | 1.20 (0.45) | 1.93 (1.18) | 2.37 (0.91) |
| T1-2 vs T3 | ＜0.001 | | |
| T1-2 vs T4 | ＜0.001 | | |
| T3 vs T4 | 0.714 | | |
| *P* Value | ＜0.001 | | |
| NIC_VP_ | 0.27 (0.06) | 0.34 (0.11) | 0.35 (0.12) |
| T1-2 vs T3 | ＜0.001 | | |
| T1-2 vs T4 | ＜0.001 | | |
| T3 vs T4 | 1.000 | | |
| *P* Value | ＜0.001 | | |
| λHU_VP_ | 2.07 (0.68) | 2.35 (0.62) | 3.09 (1.07) |
| T1-2 vs T3 | 0.180 | | |
| T1-2 vs T4 | ＜0.001 | | |
| T3 vs T4 | 0.009 | | |
| *P* Value | ＜0.001 | | |

DLSCT dual-layer detector spectral CT, pT pathological stage, Eff-Z effective atomic number, NIC normalized iodine concentration, AP arterial phase, VP venous phase, λHU slope of the spectral HU curve,

Normally distributed data were analyzed by Student’s t test or ANOVA, and were expressed as means ± standard deviations. Non-normally distributed data were analyzed by Mann-Whitney U test or Kruskal-Wallis H test, and were expressed as medians ( interquartile ranges)

## **Table 2** Pairwise comparison of ROC curves for T stages

| **Eff-Z vs NIC_AP_** | |
| --- | --- |
| Difference between areas | 0.0234 |
| Standard Error a | 0.0480 |
| 95% Confidence Interval | -0.0707 to 0.117 |
| z statistic | 0.487 |
| Significance level | P=0.6263 |
| **Eff-Z vs λHU_AP_** | |
| Difference between areas | 0.0324 |
| Standard Error a | 0.0434 |
| 95% Confidence Interval | -0.0526 to 0.117 |
| z statistic | 0.748 |
| Significance level | P=0.4544 |
| **Eff-Z vs NIC_VP_** | |
| Difference between areas | 0.0335 |
| Standard Error a | 0.0527 |
| 95% Confidence Interval | -0.0698 to 0.137 |
| z statistic | 0.635 |
| Significance level | P=0.5253 |
| **Eff-Z vs λHU_VP_** | |
| Difference between areas | 0.144 |
| Standard Error a | 0.0718 |
| 95% Confidence Interval | 0.00371 to 0.285 |
| z statistic | 2.012 |
| Significance level | P=0.0443 |
| **NIC_AP_ vs λHU_AP_** | |
| Difference between areas | 0.0558 |
| Standard Error a | 0.0390 |
| 95% Confidence Interval | -0.0207 to 0.132 |
| z statistic | 1.430 |
| Significance level | P=0.1528 |
| **NIC_AP_ vs NIC_VP_** | |
| Difference between areas | 0.0101 |
| Standard Error a | 0.0522 |
| 95% Confidence Interval | -0.0922 to 0.112 |
| z statistic | 0.194 |
| Significance level | P=0.8463 |
| **NIC_AP_ vs λHU_VP_** | |
| Difference between areas | 0.121 |
| Standard Error a | 0.0700 |
| 95% Confidence Interval | -0.0161 to 0.258 |
| z statistic | 1.730 |
| Significance level | P=0.0837 |
| **λHU_AP VS_ NIC_VP_** | |
| Difference between areas | 0.0659 |
| Standard Error a | 0.0479 |
| 95% Confidence Interval | -0.0280 to 0.160 |
| z statistic | 1.376 |
| Significance level | P=0.1687 |
| **λHU_AP_ vs λHU_VP_** | |
| Difference between areas | 0.177 |
| Standard Error a | 0.0637 |
| 95% Confidence Interval | 0.0522 to 0.302 |
| z statistic | 2.779 |
| Significance level | P=0.0054 |
| **NIC_VP_ vs λHU_VP_** | |
| Difference between areas | 0.111 |
| Standard Error a | 0.0528 |
| 95% Confidence Interval | 0.00753 to 0.214 |
| z statistic | 2.103 |
| Significance level | P=0.0355 |

Eff-Z effective atomic number, NIC normalized iodine concentration, AP arterial phase, VP venous phase, λHU slope of the spectral HU curve,

## Table 3 Pairwise comparison of ROC curves for histologic grade

| Eff-Z vs NIC_AP_ | |
| --- | --- |
| Difference between areas | 0.00758 |
| Standard Error a | 0.0500 |
| 95% Confidence Interval | -0.0904 to 0.106 |
| z statistic | 0.152 |
| Significance level | P = .8796 |
| Eff-Z vs NIC_VP_ | |
| Difference between areas | 0.169 |
| Standard Error a | 0.0522 |
| 95% Confidence Interval | 0.0668 to 0.271 |
| z statistic | 3.239 |
| Significance level | P = .0012 |
| Eff-Z vs λHU_AP_ | |
| Difference between areas | 0.00268 |
| Standard Error a | 0.0441 |
| 95% Confidence Interval | -0.0838 to 0.0892 |
| z statistic | 0.0608 |
| Significance level | P = .9515 |
| NIC_AP_ vs NIC_VP_ | |
| Difference between areas | 0.161 |
| Standard Error a | 0.0539 |
| 95% Confidence Interval | 0.0558 to 0.267 |
| z statistic | 2.996 |
| Significance level | P = .0027 |
| NIC_AP_ vs λHU_AP_ | |
| Difference between areas | 0.0103 |
| Standard Error a | 0.0389 |
| 95% Confidence Interval | -0.0659 to 0.0864 |
| z statistic | 0.264 |
| Significance level | P = .7918 |
| NIC_VP_ vs λHU_AP_ | |
| Difference between areas | 0.172 |
| Standard Error a | 0.0514 |
| 95% Confidence Interval | 0.0709 to 0.272 |
| z statistic | 3.340 |
| Significance level | P = .0008 |
